# Histological and Transcriptomic Analysis during Bulbil Formation in *Lilium lancifolium*

**DOI:** 10.3389/fpls.2017.01508

**Published:** 2017-08-30

**Authors:** Panpan Yang, Leifeng Xu, Hua Xu, Yuchao Tang, Guoren He, Yuwei Cao, Yayan Feng, Suxia Yuan, Jun Ming

**Affiliations:** ^1^College of Landscape Architecture, Nanjing Forestry University Nanjing, Jiangsu, China; ^2^The Institute of Vegetables and Flowers, Chinese Academy of Agricultural Sciences Beijing, China

**Keywords:** *Lilium lancifolium*, bulbil formation, histology, transcriptome, starch synthesis, plant hormones

## Abstract

Aerial bulbils are an important propagative organ, playing an important role in population expansion. However, the detailed gene regulatory patterns and molecular mechanism underlying bulbil formation remain unclear. Triploid *Lilium lancifolium*, which develops many aerial bulbils on the leaf axils of middle-upper stem, is a useful species for investigating bulbil formation. To investigate the mechanism of bulbil formation in triploid *L. lancifolium*, we performed histological and transcriptomic analyses using samples of leaf axils located in the upper and lower stem of triploid *L. lancifolium* during bulbil formation. Histological results indicated that the bulbils of triploid *L. lancifolium* are derived from axillary meristems that initiate *de novo* from cells on the adaxial side of the petiole base. Transcriptomic analysis generated ~650 million high-quality reads and 11,871 differentially expressed genes (DEGs). Functional analysis showed that the DEGs were significantly enriched in starch and sucrose metabolism and plant hormone signal transduction. Starch synthesis and accumulation likely promoted the initiation of upper bulbils in triploid *L. lancifolium*. Hormone-associated pathways exhibited distinct patterns of change in each sample. Auxin likely promoted the initiation of bulbils and then inhibited further bulbil formation. High biosynthesis and low degradation of cytokinin might have led to bulbil formation in the upper leaf axil. The present study achieved a global transcriptomic analysis focused on gene expression changes and pathways' enrichment during upper bulbil formation in triploid *L. lancifolium*, laying a solid foundation for future molecular studies on bulbil formation.

## Introduction

A bulbil, also known as a bulblet, consists of a small bud with a short stem (Bell and Bryan, 2008). The term also applies to aerial tubers that can be generated at the angle between a leaf and stem or in place of a flower (Ceplitis and Bengtsson, 2004). A bulbil can be released from the parent plant and grow independently into a new plant, making it an important reproductive organ (Callaghan et al., 1997). At higher elevations and in arid areas, some plants survive extreme conditions through bulbil reproduction (Steiner et al., 2012; Abraham-Juarez et al., 2015).

Bulbil formation is a rare natural phenomenon, but this character occurs in *Lilium*. The genus *Lilium* belongs to the family Liliaceae and is one of the most fascinating ornamental groups. *Lilium* species can be propagated via both sexual propagation through seeds and asexual propagation through underground bulb scales or aboveground bulbils (Bach and Sochacki, 2012). To maintain uniformity and genetic purity, most commercial cultivars are propagated by vegetative means (Maesato et al., 1991; Kumar et al., 2006). Scale-cutting propagation is an efficient method of asexual reproduction. However, scales must be manually separated from parent bulbs, and each scale can produce one or two bulblets, resulting in low propagation efficiency. As a special asexual reproductive organ, bulbils can be generated from each leaf axil (Figure S1B). In total, dozens or even hundreds of bulbils can be obtained per plant. Each mature bulbil eventually falls from the leaf axil to the ground and grows into a new plant as an individual (Suh and Roh, 2014). Consequently, bulbil propagation is more efficient and convenient than scale-cutting propagation. Elucidating the mechanisms that control bulbil formation can allow these mechanisms to be utilized to effectively improve the propagation coefficient of lilies.

In fact, not all species of the genus *Lilium* can form bulbils. *Lilium* includes ~115 species, but only four species (*L. lancifolium, L. sulphureum, L. sargentiae*, and *L. bulbiferum*) naturally form bulbils on the aboveground leaf axils of the stem (McRae, 1998; Liang and Tamura, 2000; Bach and Sochacki, 2012). Among bulbiferous species, *L. lancifolium* (Figure S1), the tiger lily, exhibits a number of special characters. This species is cultivated from cold zones to the subtropics as an ornamental plant, exhibiting strong adaptability (Liang and Tamura, 2000). *L. lancifolium* is widely cultivated in China for its edible bulbs and medicinal application (China Pharmacopoeia Committee, 2005; Yu et al., 2015), with a production value of approximately six billion Yuan per year. In addition, *L. lancifolium* is characterized by its complex ploidy. Most wild *Lilium* species are diploid (2n = 2x = 24). The only exception is *L. lancifolium*, which exhibits complex ploidy that includes both diploid and autotriploid (2n = 3x = 36), with triploids accounting for the majority of its distribution (Noda, 1986; Kim et al., 2006; Chung et al., 2015). The triploids are completely sterile, and *L. lancifolium* therefore cannot extend its population through seeds (Noda, 1986). However, triploid *L. lancifolium* produces large numbers of purple-black bulbils on the leaf axils along the stem, making bulbil propagation a significant asexual strategy for triploid forms of *L. lancifolium* (Suzuki and Yamagishi, 2016). An interesting phenomenon in this species is that bulbils are exclusively generated from the leaf axils in the middle-upper portion of the stem. Thus, it is interesting and meaningful to study the mechanisms of bulbil formation in triploid *L. lancifolium*.

Bulbil formation has been studied in several plant species, such as *Dioscorea batatas, Allium sativum, Titanotrichum oldhamii, Lilium sulphureum, Pinellia ternata*, and *Agave tequilana*. Histologic studies of bulbiferous species have shown that aerial bulbils are derived from axillary meristems (AMs). The process of axillary bulbil formation includes two steps: (1) meristematic cells in the leaf axil divide continuously to form bulbil primordia; and (2) the bulbil primordia grow and differentiate to form the bulbil structure (Zhang et al., 1994; Wang and Cronk, 2003; Li et al., 2012; Luo et al., 2014). It is widely recognized that plant hormones, particularly auxin (IAA), play important roles in the above processes. In *Agave tequilana*, removal of flower buds results in a reduction in IAA flux in pedicel vascular tissue, stimulating the development of new meristems and vegetative bulbils (Abraham-Juarez et al., 2015). Conversely, the application of exogenous IAA to cut pedicel tissue suppresses bulbil formation at the bracteoles, indicating an inhibitory effect of IAA on axillary bulbils. In addition, cytokinin (CK) stimulates the formation of axillary tubers, whereas gibberellin (GA) and strigolactone (SL) inhibit their formation in potato (Navarro et al., 2015).

At the molecular level, the Gesneriaceae *FLO/LFY* homolog (*GFLO*) is involved in the regulation of bulbil formation in *Titanotrichum oldhamii*, and its expression is down-regulated with bulbil development (Wang et al., 2004). In *Agave tequilana*, the expression of two class I *KNOX* genes (*AtqKNOX1* and *AtqKNOX2*) occurs at bulbil initiation and increases as bulbils mature (Abraham-Juarez et al., 2010). The *AtqMADS1, 2, 4, 6*, and *7* genes all present the same pattern of reduced expression during bulbil development in *Agave tequilana* (Sandoval et al., 2012). Expression of the *LONELY GUY* (*LOG1*) gene confers the ability for axillary tomato meristems to undergo *de novo* formation of tuber-like organs (Eviatar-Ribak et al., 2013). Silencing of the SL biosynthetic *StCCD8* gene in potato results in the formation of aerial tubers (Pasare et al., 2013). Although several genes related to bulbil formation have been isolated, the metabolic pathways and detailed regulatory patterns involved in the process of bulbil formation remain unclear.

RNA sequencing (RNA-Seq) is a recently developed, effective and powerful tool for analyzing the entire transcriptome composition and the gene expression patterns of various biological processes, particularly in non-model organisms for which genomic sequences are not available (Wang et al., 2009). To date, RNA-Seq technology has been successfully applied for the study of flower color (Xu et al., 2017), flowering (Liu et al., 2014), bulblet development (Li et al., 2014), growth characteristics (Zhu et al., 2016), and stress responses mechanisms (Wang et al., 2014a,b) of the lily. No detailed analysis has previously been performed on transcriptional changes during bulbil formation in *Lilium*.

To understand the process of bulbil formation in triploid *L. lancifolium*, we first performed a histological analysis of leaf axil samples from the upper and lower stem throughout the period of bulbil formation and evaluated developmental events. Using the Illumina HiSeq 4000 platform, we then performed *de novo* transcriptome sequencing of three types of leaf axil samples from the upper stem (axil, swelling axil and white dot structure axil) and one type of leaf axil sample from the lower stem (at the stage of swelling in the upper axil). This work provides comprehensive transcript information and gene expression data on bulbil initiation and formation, laying a solid foundation for future molecular studies on bulbil formation.

## Materials and methods

### Plant materials

**Figure 1:**
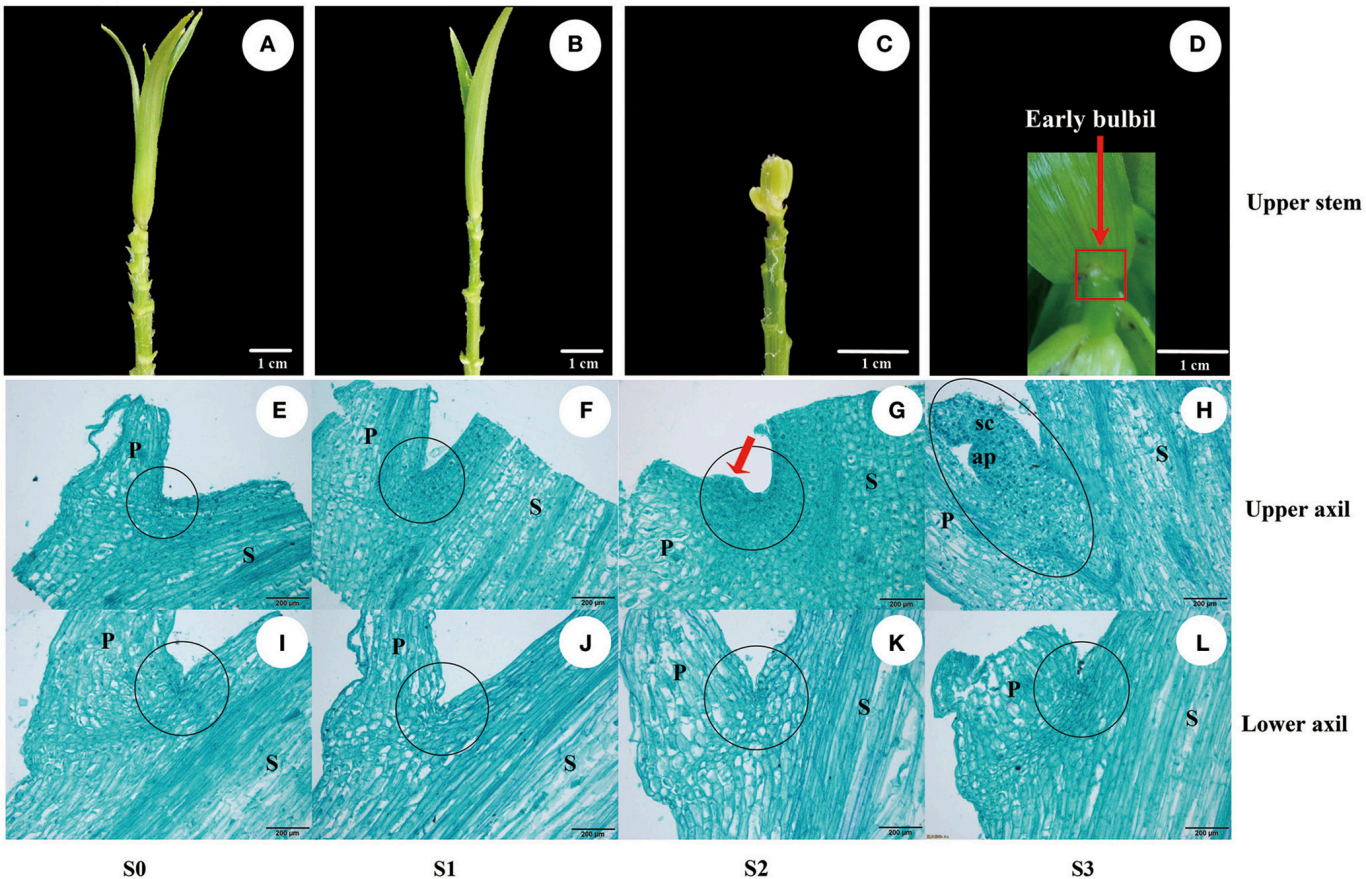
Bulbil formation in *L*. *lancifolium*. **(A–D)** Stages of bulbil formation in the upper leaf axil: **(A,B)** S0 and S1 stages exhibiting apparent upper internodes; **(C)** S2 stage exhibiting swelling; **(D)** S3 stage exhibiting a white dot structure (early bulbil). **(E–L)** Microscopy images of paraffin-embedded samples of leaf axils located on the upper **(E–H)** and lower stem **(I–L)** from four stages (S0–S3). Scale bar for samples images = 1 cm; Scale bar for microscopy images = 200 μm. P, Petiole; S, stem; sc, scale; ap, apical point; black circle, leaf axil.

The autotriploid species *Lilium lancifolium* was used as the material in this study. *L. lancifolium* bulbs of uniform size with 1-2-cm buds were grown in soil in a greenhouse at the Institute of Vegetables and Flowers, Chinese Academy of Agricultural Sciences, Beijing, China, in early June of 2015. Samples of the upper and lower sectional stem nodes with ~0.4-mm petioles (Figure S2) were collected by hand-dissection with a double-edge blade every 5 days during the observation period, from the appearance of upper internodes (1 month after planting) to the appearance of white dot structures (early bulbils), producing four stages (S0, S1, S2, and S3) (Figures 1A–D), and these samples were used for histological analysis. The leaf axil zone samples (including leaf axil tissue, partial stem node and ~0.4-mm petiole) (Figure S2) from the upper stem at stage 1 (U1), stage 2 (U2), and stage 3 (U3) and from the lower stem at stage 2 (D2) were cut by hand with a disposable double-edge blade. All leaf axil samples were immediately frozen in liquid nitrogen and stored at −80 °C until further RNA-Seq and quantitative real-time PCR analyses. Three biological replicates for each sample were collected randomly from five individual plants, and each biological replicate contained five to seven leaf axils.

### Histological analysis

Samples of the upper and lower sectional stem nodes with ~0.4-mm petioles (Figure S2) from each developmental stage were fixed in FAA fixation buffer (formaldehyde: glacial acetic acid: 70% ethanol, 1:1:18) for 48 h at 4°C. The fixed samples were dehydrated in a graded series of ethanol (70%, 85%, 95% and 100%), followed by a xylene/ethanol series (xylene: ethanol 1:2, 1:1, and 2:1 and 100% xylene). Xylene was gradually replaced with paraffin (melting point of 58°C–60°C) at 60°C for 3 days. Sections (10 μm thick) were obtained using a rotary microtome and were double-stained with 5% (w/v) Safranine T and 0.5% (w/v) Fast Green FCF.

### RNA extraction

Total RNA was extracted using the RNAprep Pure Plant Kit (Polysaccharide- & Polyphenolic-rich) (TIANGEN, Beijing, China) according to the manufacturer's instructions, and DNA contamination was removed with RNase-free DNase I. RNA degradation and contamination were monitored using 1% agarose gels. RNA purity and concentration were measured using a NanoPhotometer® spectrophotometer (IMPLEN, CA, USA) and Qubit® RNA Assay Kit with a Qubit® 2.0 Fluorometer (Life Technologies, CA, USA), respectively. RNA integrity (RIN) was assessed using the RNA Nano 6000 Assay Kit with the Agilent Bioanalyzer 2100 system (Agilent Technologies, CA, USA). RNA samples with RINs ≥ 8.0, A260/280 ranging from 1.8 to 2.1, and 25S:18S ranging from 1.7 to 2.0 were used for library preparation. Three biological replicates were used for RNA extraction and further RNA-Seq.

### cDNA library construction and sequencing

Twelve cDNA libraries (three replicates for each sample) were constructed using the NEBNext® Ultra™RNA Library Prep Kit for Illumina® (NEB, USA) following the manufacturer's protocol. Briefly, mRNA was purified from 1.5 μg of total RNA using oligo (dT) magnetic beads, and then broken into short fragments of 300–500 bp by adding fragmentation buffer. First-strand cDNA was synthesized using random hexamer primer. Second-strand cDNA synthesis was subsequently performed using RNase H and DNA polymerase I. After adenylation of the 3′ ends of cDNA fragments, NEBNext adapter oligonucleotides were ligated to prepare for hybridization, and then the cDNA fragments were purified using AMPure XP system (Beckman Coulter, Beverly, USA) to select the fragments of preferentially 150–200 bp in length. Then 3 μL of USER Enzyme (NEB, USA) was used with size-selected and adaptor-ligated cDNA fragments. Then PCR was performed with Phusion High-Fidelity DNA polymerase, universal PCR primers and Index(X) Primer. PCR products were purified with the AMPure XP system, and library quality was assessed using the Agilent Bioanalyzer 2100 system. Finally, all cDNA libraries were sequenced on the Illumina HiSeq 4000 platform using the paired-end technology by Novogene Co. (Beijing, China), and 150-bp paired-end reads were generated.

### *De novo* transcriptome assembly

Clean reads were obtained from the sequencing reads by removing reads containing adapters, reads with more than 10% unknown nucleotides, and low-quality reads. The Q20, Q30, GC content and sequence duplication levels were calculated from the clean data. Then, *de novo* assembly based on the clean data was performed using the Trinity program (Grabherr et al., 2011). Trinity includes three independent software modules: Inchworm, Chrysalis and Butterfly. Short reads with overlapping sequences are first assembled by Inchworm to form longer transcripts, known as contigs. Chrysalis then clusters the contigs into clusters and constructs complete *de Bruijn* graphs for each cluster. Finally, Butterfly reports the full-length transcripts. The final sequences of Trinity assembly were defined as unigenes.

### Functional annotation and classification of unigenes

All unigene sequences were aligned to Nt nucleotide databases using Blastn and were aligned by Blastx to protein databases such as the NCBI non-redundant (Nr) database (http://www.ncbi.nlm.nih.gov), the Swiss-Prot protein database (http://www.ebi.ac.uk/uniprot), the Pfam database (http://pfam.sanger.ac.uk), the KEGG database (http://www.genome.jp/kegg/) (Kanehisa and Goto, 2000), and the KOG/COG database (http://www.ncbi.nlm.nih.gov/COG). Each unigene was functionally annotated based on the protein sharing the highest sequence similarity with the given unigene. Gene Ontology (GO, http://www.geneontology.org/) annotation was performed using Blast2GO software (version 2.5) (Gotz et al., 2008) based on the results of Nr and Pfam annotation. Web Gene Ontology Annotation Plot (WEGO, http://wego.genomics.org.cn/cgi-bin/wego/index.pl) was then employed to produce GO functional classifications (Ye et al., 2006).

### Analysis of differential gene expression

The FPKM (fragments per kilobase per million base pairs) method was used to calculate the expression of unigenes. Then, differential expression analysis of two groups was performed using the DESeq R package (1.10.1). The resulting *p-value* was adjusted using the Benjamini and Hochberg's approach (Benjamini and Hochberg, 1995) for controlling the false discovery rate (FDR). The DEGs between two samples were determined based on an adjusted *p-value* (*q-value*) < 0.05 and |log2Foldchange| ≥ 1. GO enrichment analysis (corrected *p* < 0.05) of the DEGs was implemented with the GOseq R package-based Wallenius noncentral hypergeometric distribution (Young et al., 2010). KOBAS software was used to test the statistical enrichment of DEGs in KEGG pathways (Mao et al., 2005). KEGG pathways with a threshold *q* < 0.05 were considered significantly enriched in the DEGs.

### Quantitative real-time PCR validation and expression analysis

Sixteen DEGs were selected for RNA-Seq data validation and expression analysis using quantitative real-time PCR (qRT-PCR). For cDNA synthesis, 500 ng of total RNA (without DNA contamination) was reverse-transcribed using the TransScript^®;^ One-Step gDNA Removal and cDNA Synthesis SuperMix kit (TRAN, Beijing, China). Specific primers for qRT-PCR were designed with Primer 5.0 software, and the detailed information is listed in Table S1. All reactions were performed in 96-well plates using the CFX96 Real-Time System (Bio-Rad, USA). Each PCR reaction (20 μL) contained 2 μL of cDNA (1:3 dilution), 0.5 μL of each forward and reverse primer (10 μM), 10 μL of 2 × SYBR® Premix Ex Taq™ (Tli RNaseH Plus) (Takara) and 7 μL of ddH_2_O. Amplification reactions were performed under the following conditions: 95°C for 3 min, followed by 40 cycles of 95°C for 20 s, 60°C for 10 s, and 72°C for 20 s. A melting curve was generated for each PCR reaction to determine the amplification specificity and the presence of reaction contaminations. The melting curve was obtained by heating the amplification products from 60°C to 95°C in 5-s intervals. The primer efficiency was analyzed with CFX Manager™Software v3.1 (Bio-Rad). All qRT-PCR experiments were repeated with three biological and three technical replicates. The *lilyActin* (Gene Bank Accession Number: JX826390) gene (forward primer: 5′-GCATCACACCTTCTACAACG-3′; reverse primer: 5′-GAAGAGCATAACCCTCATAGA-3′) was used as the internal control for normalization, and the relative expression levels of the target genes were calculated using the 2^−ΔΔCt^ method against the internal control (Livak and Schmittgen, 2001).

### Soluble sugar and starch content measurement

Soluble sugar and starch were measured using an anthrone colorimetric method. Briefly, samples of leaf axils from the upper and lower stem at different stages were ground into powder using liquid nitrogen. Then, 0.2 g of powder was weighed in a 10-mL centrifuge tube, to which 6 mL of distilled water was added. The mixed homogenate was then incubated in boiling water for 30 min. Then, the extracted solution was centrifuged at 5,000 rpm for 10 min. The supernatant was collected, and the extraction was repeated three times (5,000 rpm for 10 min each). All supernatants were collected into 25-mL volumetric flasks for the determination of soluble sugar. The residual was re-dissolved in 20 mL of distilled water and incubated in boiling water for 15 min. Then, 2 mL of cold 9.2 mol/L perchloric acid was added, and the mixture was again incubated in boiling water for 15 min. The solution was finally filtered in a 50-mL volumetric flask, and the filtrate was used for the determination of starch. The soluble sugar and starch contents were measured by their absorption values at 630 nm and 485 nm, respectively, and calibration curves were constructed with standard solutions of sucrose and soluble starch, respectively. Three biological replicates for each sample and three technical replicates for each biological replicate were performed.

### Determination of IAA concentration

Samples of leaf axils from the upper and lower stem at different stages were ground into powder using liquid nitrogen. Then, 0.2 g of powder was weighed in a 10-mL centrifuge tube and extracted overnight at 4°C in cold 80% (v/v) methanol containing 1 mM butylated hydroxytoluene. The extracts were collected after centrifugation at 10,000 × g at 4°C for 20 min and were then passed through a C18 Sep-Pak cartridge (Waters). The efflux was dried with nitrogen (N_2_), and the IAA content was determined by means of enzyme-linked immunosorbent assay (ELISA) (He, 1993; Yang et al., 2001). Three biological replicates for each sample and three technical replicates for each biological replicate were performed.

### Statistical analysis

The data for soluble sugar, starch and endogenous IAA contents and the relative expression analyses of genes were presented as the means with standard errors from three biological replicates. All statistical analyses were performed using SAS 9.1 (SAS Institute, Cary, NC, USA), and statistical significance was defined at *p* ≤ 0.05. Non-overlapping letters (a-d) indicated significant differences between the comparisons, based on the ANOVA analysis and Duncan's multiple range test.

## Results

### Morphological and histological description of bulbil formation in triploid *L. lancifolium*

To describe the process of bulbil formation in triploid *L. lancifolium*, the upper and lower sectional stem nodes with ~0.4-mm petioles (Figure S2) were collected at each developmental stage (S0–S3). The process of bulbil formation was observed on leaf axils of the upper stem (Figures 1A–D), and a defined early bulbil structure (white dot structure) was discovered in the upper axil at stage S3 (Figure 1D: red arrow). Based on morphological and histological observations, no morphological changes in the upper leaf axil were detectable at stage S0 or S1 (Figures 1E,F). At stage S2, the adaxial side of the petiole base in the upper stem showed obvious bulging (Figure 1G: red arrow). Cells in the swelling region exhibited large nuclei and dense protoplasm. They were small and serried, whereas the relative size of the cells was larger outside of this region (Figure 1G). At stage S3, actively dividing cells differentiated to form the bulbil structure (Figure 1H) on the upper leaf axil. In contrast, there were no obvious structural changes in the leaf axils of the lower stem (Figures 1I–L). Visual analyses of sections suggested that the bulbils originated from several layers of parenchyma cells in the leaf axil at stage S2. In addition, upper axil swelling was found to be a key stage for initiating bulbil formation.

### Transcriptome sequencing and assembly

To obtain a comprehensive overview of the triploid *L. lancifolium* transcriptome during bulbil initiation and formation, leaf axils on the upper stem at stages S1, S2 and S3 (U1, U2, and U3) and leaf axils showing no bulbil formation on the lower stem at stage S2 (D2) were obtained for an RNA-Seq analysis with three biological replicates. Sequencing of cDNA libraries from the twelve samples resulted in 46–70 million raw paired-end reads with greater than 6.5 Gb per sample (Table 1). All raw reads were deposited in the NCBI Sequence Reads Archive (SRA) under the accession number SRP103184. Given the lack of reference genome sequences for lilies, a *de novo* assembly strategy was used to construct transcripts. After ambiguous nucleotides, low-quality sequences and contaminated sequences were removed, ~45–67 million clean reads were obtained for assembly from each library. For all the sequence data, the Q20 and Q30 percentages were greater than 94 and 86%, respectively (Table 1). A total of 598,060,858 clean reads obtained from 649,714,466 raw reads (92.05%) were used in the assembly, and 389,636 transcripts (contigs) were generated, with a mean length of 674 bp and an N50 of 1105 bp (Tables S2, S3, Figure S3). All contigs were assembled into 293,858 non-redundant unigenes with an average length of 549 bp and an N50 of 752 bp (Tables S2, S3, Figure S3). All unigenes were longer than 200 bp. In total, 211,581 of the unigenes (72%) were 200–500 bp in length, and 11,337 (3.90%) were longer than 2 kb (Tables S3).

**Table 1:** Summary of RNA-Seq data from leaf axils in the upper (U) and lower (D) stems of *L. lancifolium*.

**Sample**	**Raw reads**	**Clean reads**	**Clean bases**	**Error (%)**	**Q20 (%)**	**Q30 (%)**	**GC (%)**
U1-1	58,224,342	48,368,422	7.26G	0.03	95.64	87.86	49.39
U1-2	47,095,472	45,374,644	6.81G	0.03	94.72	87.56	50.83
U1-3	56,009,802	46,678,280	7.00G	0.03	95.62	87.87	49.83
U2-1	55,315,794	46,736,626	7.01G	0.03	95.72	88.18	51.13
U2-2	48,475,732	46,692,406	7.00G	0.02	95.55	89.35	51.37
U2-3	50,120,502	48,701,208	7.31G	0.02	95.26	88.85	50.03
U3-1	69,548,006	66,545,740	9.98G	0.02	95.50	89.33	49.12
U3-2	56,517,258	46,921,990	7.04G	0.03	95.78	88.33	49.22
U3-3	52,072,828	50,155,980	7.52G	0.02	95.23	88.72	49.41
D2-1	49,055,962	47,988,428	7.20G	0.03	94.53	86.45	49.57
D2-2	60,296,838	58,890,574	8.83G	0.02	95.37	88.48	49.20
D2-3	46,981,930	45,006,560	6.75G	0.02	95.80	89.26	50.27

To assess the quality of the assembled transcripts, the clean reads were mapped back to the assembly. The results showed that 75% of the clean reads could be mapped back to the assembled transcripts, and more than 68.4% of the paired reads of each library could be mapped (Table S4). These data revealed that the quality of transcriptome sequencing and assembly was sufficiently high for further analysis.

### Functional annotation and classification of unigenes

The 293,858 unigenes were annotated via alignment with seven public databases (Table 2). The results showed that 79,950 (27.20%), 53,597 (18.23%), 63,967 (21.76%), 64,874 (22.07%), 67,672 (23.02%), and 36,214 (12.32%) unigenes were matched in the Nr, Nt, Swiss-Prot, Pfam, Gene Ontology (GO) and Clusters of Orthologous Groups of proteins (KOG/COG) databases, respectively. In total, 34,491 unigenes (11.73%) were annotated in the Kyoto Encyclopedia of Genes and Genomes (KEGG) database, and 112,185 unigenes (38.17%) were successfully annotated in at least one of the seven databases, among which 15,569 unigenes (5.29%) were simultaneously annotated in all databases.

**Table 2:** Annotation of assembled unigenes against seven public databases.

**Annotation database**	**Number of annotated unigenes**	**Percentage (%)**
NR	79,950	27.20
NT	53,597	18.23
SwissProt	63,967	21.76
KEGG	34,491	11.73
PFAM	64,874	22.07
GO	67,672	23.02
KOG	36,214	12.32
Annotated in all databases	15,569	5.29
Annotated in at least one database	112,185	38.17
Total unigenes	293,858	100

GO assignment was performed to classify the functions of the predicted unigenes. The 67,672 unigenes annotated in the GO database were categorized into 56 functional groups and divided into three main ontologies: biological process (BP), cellular component (CC), and molecular function (MF) (Figure S4). Regarding the MF category, genes involved in “binding” (51.82%) and “catalytic activity” (42.87%) accounted for the largest proportion. Within the CC category, the “cell” (31.14%), “cell part” (31.13%), “organelle” (20.33%) and “macromolecular complex” (19.73%) categories were highly represented. Moreover, “cellular processes” (55.00%) and “metabolic processes” (52.51%) were dominant in the BP category (Table S5, Figure S4).

To further evaluate the functions of all assembled unigenes, we performed a search against the KOG/COG database. Based on sequence homology, 36,214 unigenes were assigned to a KOG functional classification, divided into 26 specific categories. Among these categories, the “general functional prediction only” (16.22%) cluster represented the largest category, followed by “posttranslational modification, protein turnover, chaperones” (13.26%), “translation, ribosomal structure and biogenesis” (9.81%), “signal transduction mechanisms” (9.54%), “energy production and conversion” (6.01%) and “intracellular trafficking, secretion, and vesicular transport” (5.98%). The “cell motility” (0.10%) and “unnamed protein” (0.01%) clusters were the smallest categories (Table S6).

KEGG pathways were also searched to allow a biological interpretation of the functions of the assembled unigenes. According to the KEGG results, 34,491 unigenes were mapped to predicted metabolic pathways (Figure S5, Table S7), and the majority of unigenes were classified into pathways related to translation (11.32%), carbohydrate metabolism (8.36%), folding, sorting and degradation (8.12%). All the pathways were divided into five categories, with “metabolism” (42.90%) representing the largest category (Figure S5D class). Within “metabolism,” carbohydrate metabolism was the most represented category; within “carbohydrate metabolism,” starch and sucrose metabolism was most dominant (Table S7).

### Analysis of differentially expressed genes (DEGs)

#### Verification of RNA-Seq data via qRT-PCR

**Figure 2:**
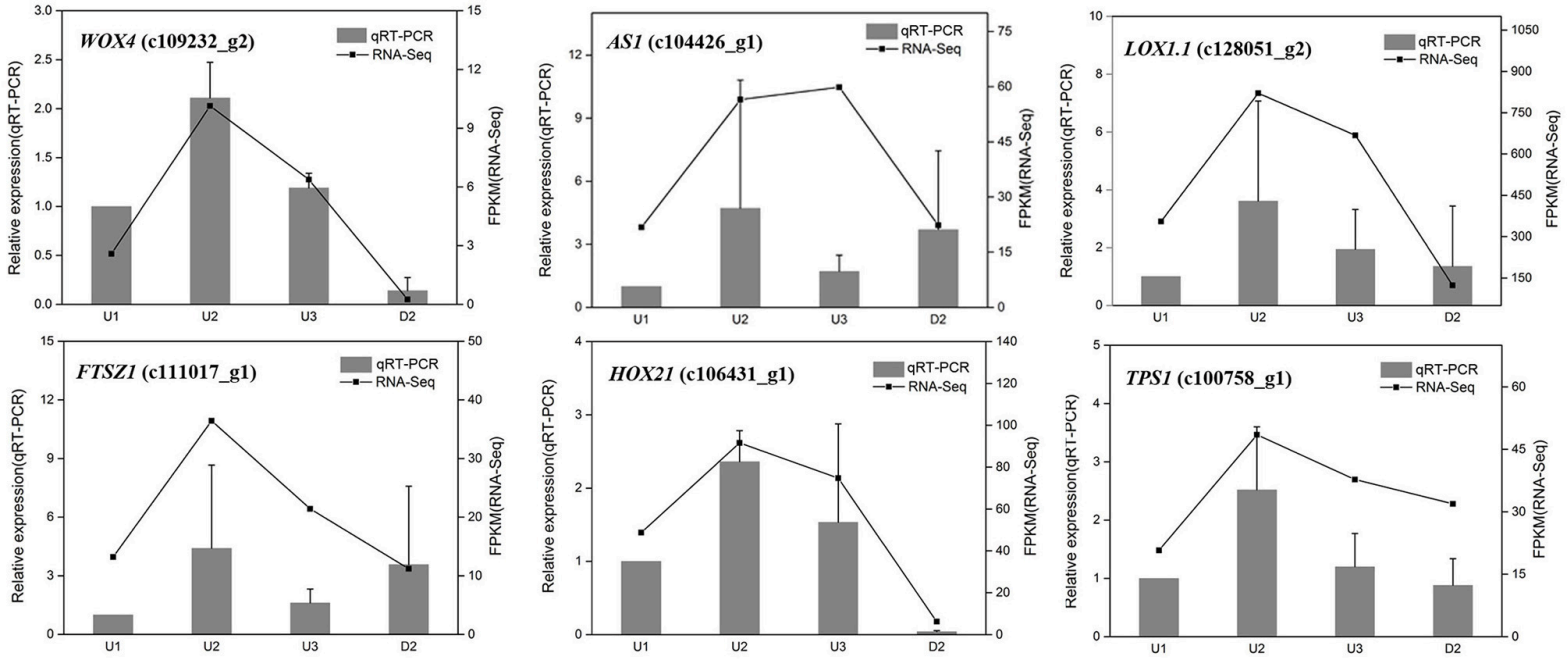
qRT-PCR validation of differential gene expression in U1, U2, U3, and D2. The left y-axis indicates relative gene expression levels. The right y-axis indicates gene expression levels calculated based on mean FPKM value of each sample.

To validate the differential expression by RNA-Seq, sixteen unigenes were selected from the identified DEGs for qRT-PCR analysis, including six randomly selected genes (*WOX4*_c109232_g2, *AS1_*c104426_g1, *LOX1.1_*c128051_g2, *FTSZ1_*c111017_g1, *HOX21*_c106431_g1, and *TPS1_*c100758_g1), six genes related to starch biosynthesis (*SUS2*_c111432_g1, *UGP2*_c123358_g1, *AGPS1*_c121621_g2, *SSS3*_ c122034_g2, *WAXY*_c123636_g2, and *SBE1*_ c97206_g1) and four genes involved in auxin signaling (*YUC*_c111323_g1, *YUC10*_c128166_g2, *IAA17*_c115342_g1, and *PIN1B*_c108190_g1). The result showed that most of the expression patterns revealed by qRT-PCR were in good agreement with the RNA-Seq results, indicating the reliability of the RNA-Seq data (Figures 2, **4C**, **6D**).

#### Global analysis of DEGs

**Figure 3:**
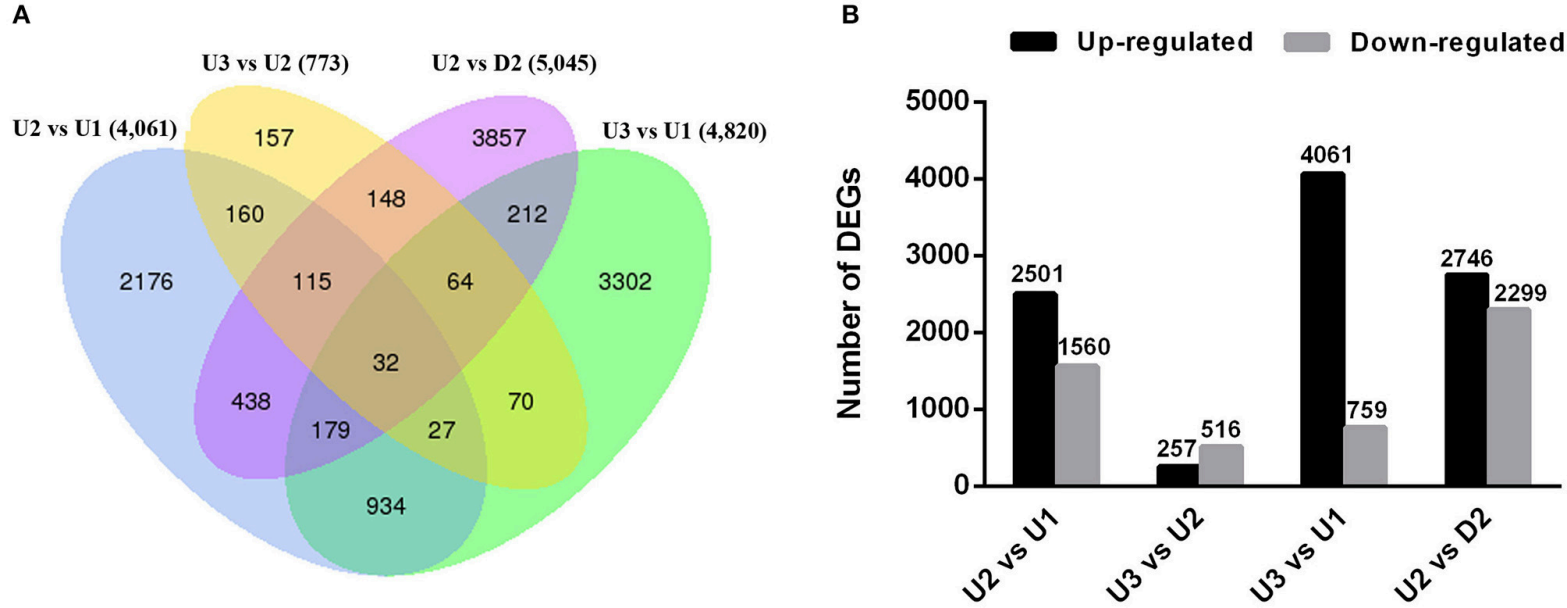
Overview of numbers of DEGs in pairwise comparisons. **(A)** Venn diagram of DEGs among the four comparisons performed (U2 vs. U1, U3 vs. U2, U2 vs. D2 and U3 vs. U1). **(B)** Histogram showing the number of DEGs that were up- or down-regulated between different libraries. The black columns indicate up-regulated DEGs, and the gray columns represent down-regulated DEGs in the four pairwise comparisons.

We sought to identify genes exhibiting significant changes in expression levels during bulbil formation in triploid *L. lancifolium*. In total, 11,871 DEGs were identified by comparing the four libraries in pairs (Table S8). In total, 4,061, 4,820, and 5,045 of the DEGs were differentially expressed between U2 vs. U1, U3 vs. U1, and U2 vs. D2, respectively (Figure 3A). The smallest difference existed between U3 and U2, with only 257 up-regulated and 516 down-regulated unigenes identified (Figure 3B). 2,176, 157, 3,302, and 3,857 DEGs were specific for U2 vs. U1, U3 vs. U2, U3 vs. U1 and U2 vs. D2, respectively (Figure 3A). Moreover, the principal component analysis (PCA) of the RNA-Seq data was performed based on the FRPM values of three replicates for each sample, and U2 and U3 groups were clustered in the same quadrant (Figure S6). The results suggested that U3 is developmentally close to U2, and significant differential gene expression was observed in the initial stage (U2) of bulbil formation. In addition, more genes were up-regulated than down-regulated in U2 vs. U1, U3 vs. U1 and U2 vs. D2 (Figure 3B), indicating that the initiation process was likely caused by the expression of up-regulated genes.

GO enrichment analysis for the DEGs was performed to classify the functions of the DEGs in pairwise of upper leaf axils at different stages during bulbil formation (U1, U2, and U3) and in pairwise of U2 and D2. The DEGs between U2 and U1, U3 and U2, U3 and U1, and U2 and D2 were assigned to 34, 10, 104, and 108 GO terms based on “biological process (BP),” “cellular component (CC),” and “molecular function” (Table S9). In all comparisons, DEGs were mainly enriched for “biological process (BP)” and were significantly enriched (corrected *p* < 0.05) for “metabolic process” in “biological process (BP).” To further explore the detailed metabolic pathways, all DEGs were mapped to terms in the KEGG database, and a KEGG pathway enrichment analysis was subsequently performed. In total, 4,061 DEGs (U2 vs. U1) were assigned to 115 pathways; 773 DEGs (U3 vs. U2) to 71 pathways; 4,820 DEGs (U3 vs. U1) to 113 pathways; and 5,045 DEGs (U2 vs. D2) to 110 pathways (Table S10). Pathway enrichment analysis revealed that the initiation and formation of bulbils involved the integration of several pathways. A *q* < 0.05 was used as the thresholds to determine significant enrichment. The annotated changes between U2 and U1 were mainly involved in starch and sucrose metabolism, amino sugar and nucleotide sugar metabolism, and phenylpropanoid biosynthesis (Figure S7A). The annotated changes between U3 and U2 were mainly involved in photosynthesis, protein processing in the endoplasmic reticulum, amino sugar and nucleotide sugar metabolism (Figure S7B). The annotated changes between U2 and D2 were mainly involved in phenylpropanoid biosynthesis, starch and sucrose metabolism, and plant hormone signal transduction (Figure S7C).

#### DEGs involved in starch and sucrose metabolism

**Figure 4:**
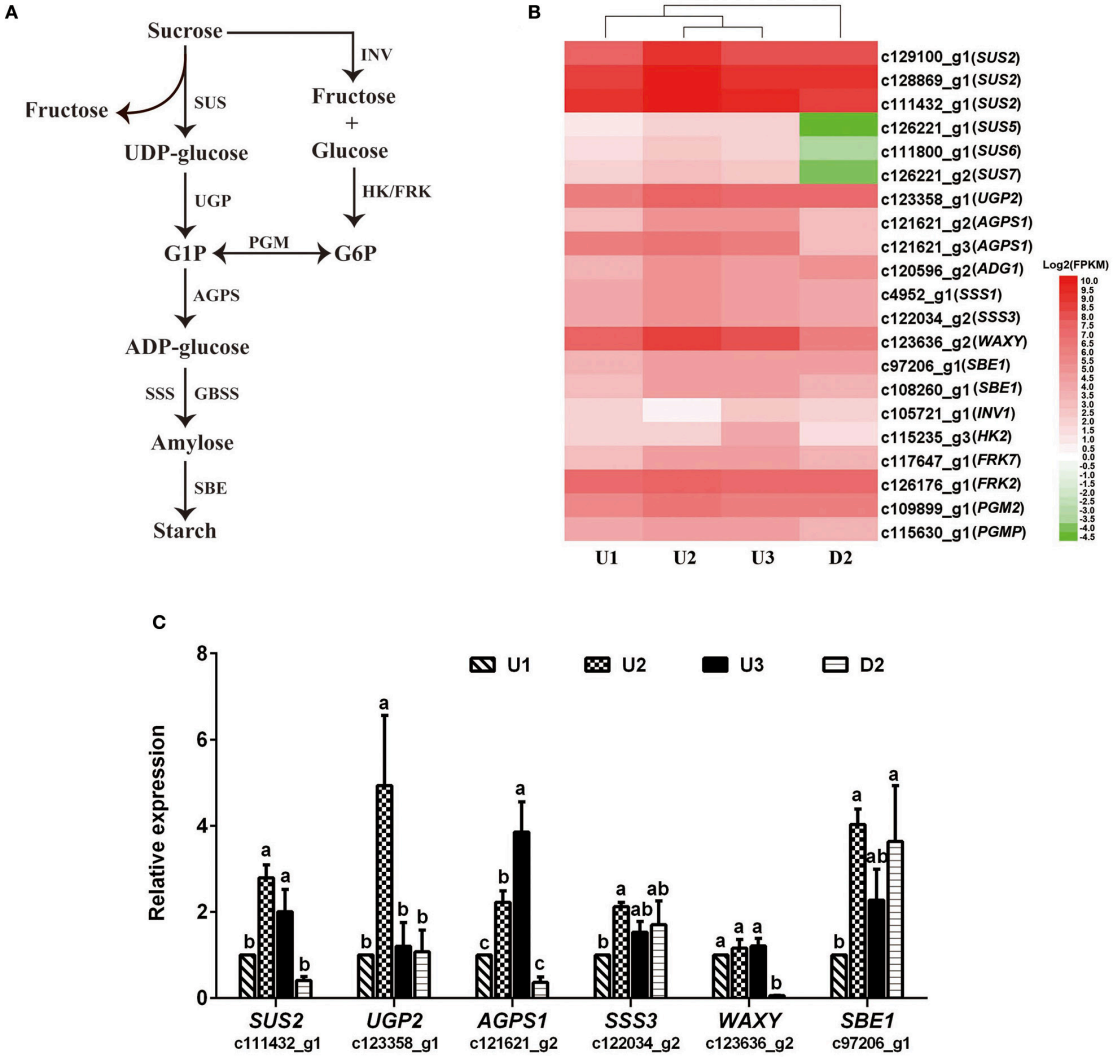
Expression patterns of DEGs assigned to starch biosynthesis and qRT-PCR analysis. **(A)** The starch biosynthesis pathway shown in this figure was modified from Yang et al. (2015). **(B)** Heatmap of the expressed genes. The FPKM values of the unigenes were log_2_ transformed, and a heatmap was generated using HemI 1.0. Red and green indicate up- and down-regulated transcripts, respectively. SUS, Sucrose synthase; UGP, UTP-glucose-1-phosphate uridylyltransferase; AGPS, glucose-1-phosphate adenylyltransferase; SSS, soluble starch synthase; GBSS, granule-bound starch synthase; SBE, starch branching enzyme; INV, beta-fructofuranosidase; HK, hexokinase; FRK, fructokinase; PGM, phosphoglucomutase. **(C)** qRT-PCR analysis of the expression profiles of six genes involved in starch biosynthesis. The x-axis represents different samples, and the y-axis shows relative gene expression levels. Columns and error bars indicate means and standard deviations, respectively. Non-overlapping letters (a-c) indicate significant differences (*p* < 0.05) between the comparisons of different samples.

**Figure 5:**
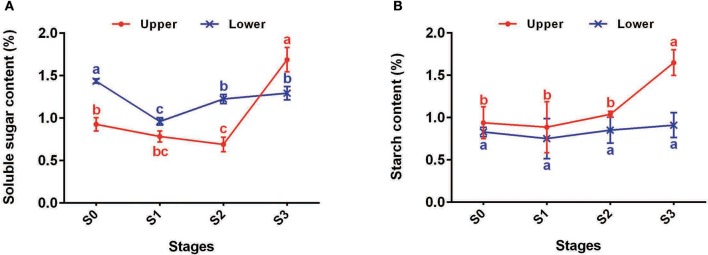
Changes of starch and sucrose contents of upper (red) and lower (blue) axils at different stages during bulbil formation. **(A)** Soluble sugar contents of upper and lower axils at different stages during bulbil formation. **(B)** Starch contents of upper and lower axils at different stages during bulbil formation. The x-axis represents different stages, and the y-axis shows content. Lines and error bars indicate means and standard deviations, respectively. Non-overlapping letters indicate significant differences (*p* < 0.05) for the different stages.

In total, 43, 8, 26, and 56 DEGs between U2 vs. U1, U3 vs. U2, U3 vs. U1, and U2 vs. D2, respectively, were enriched in starch and sucrose metabolism pathways (Ko00500) (Table S10). Most of the DEGs were up-regulated during the process of bulbil initiation and formation in the upper leaf axil in *L. lancifolium*. Among starch and sucrose metabolism genes, genes encoding starch synthases, such as sucrose synthase *(SUS)* (c129100_g1, c128869_g1, c111432_g1, c126221_g1, c111800_g1 and c126221_g2), *HK* (c115235_g3), *PGM* (c115630_g1), *UGP* (c123358_g1), *AGPS* (c121621_g2), *SSS* (c4952_g1 and c122034_g2), *GBSS* (c123636_g2), and *SBE* (c97206_g1 and c108260_g1), exhibited high expression levels during upper bulbil initiation (up-regulated in U2 vs. U1), which declined slightly in U3 (Figure 4B, Table S11). Additionally, the above DEGs were significantly up-regulated in U2 compared with D2. These results suggested that starch biosynthesis was likely predominant in bulbil initiation. Validation of *SUS2* (c111432_g1), *UGP2* (c123358_g1), *AGPS1* (c121621_g2), *SSS3* (c122034_g2), *WAXY* (c123636_g2), and *SBE1* (c97206_g1) via qRT-PCR further confirmed our observations (Figure 4C). In addition, we analyzed changes in soluble sugar content and starch content during bulbil formation in triploid *L. lancifolium* using an anthrone colorimetric method. During the process of bulbil formation in the upper portion of stem, the starch content increased slowly (from 0.94 to 1.04%) from stage S0 to S2, followed by a rapid increase to 1.65% at S3 (Figure 5B). In contrast, the soluble sugar content dropped from S0 to S2 and then increased in S3 (Figure 5A). During this process, the starch content of lower axils was less than that of the upper axils and exhibited minimal change (Figure 5B).

#### DEGs involved in plant hormone signal transduction

**Figure 6:**
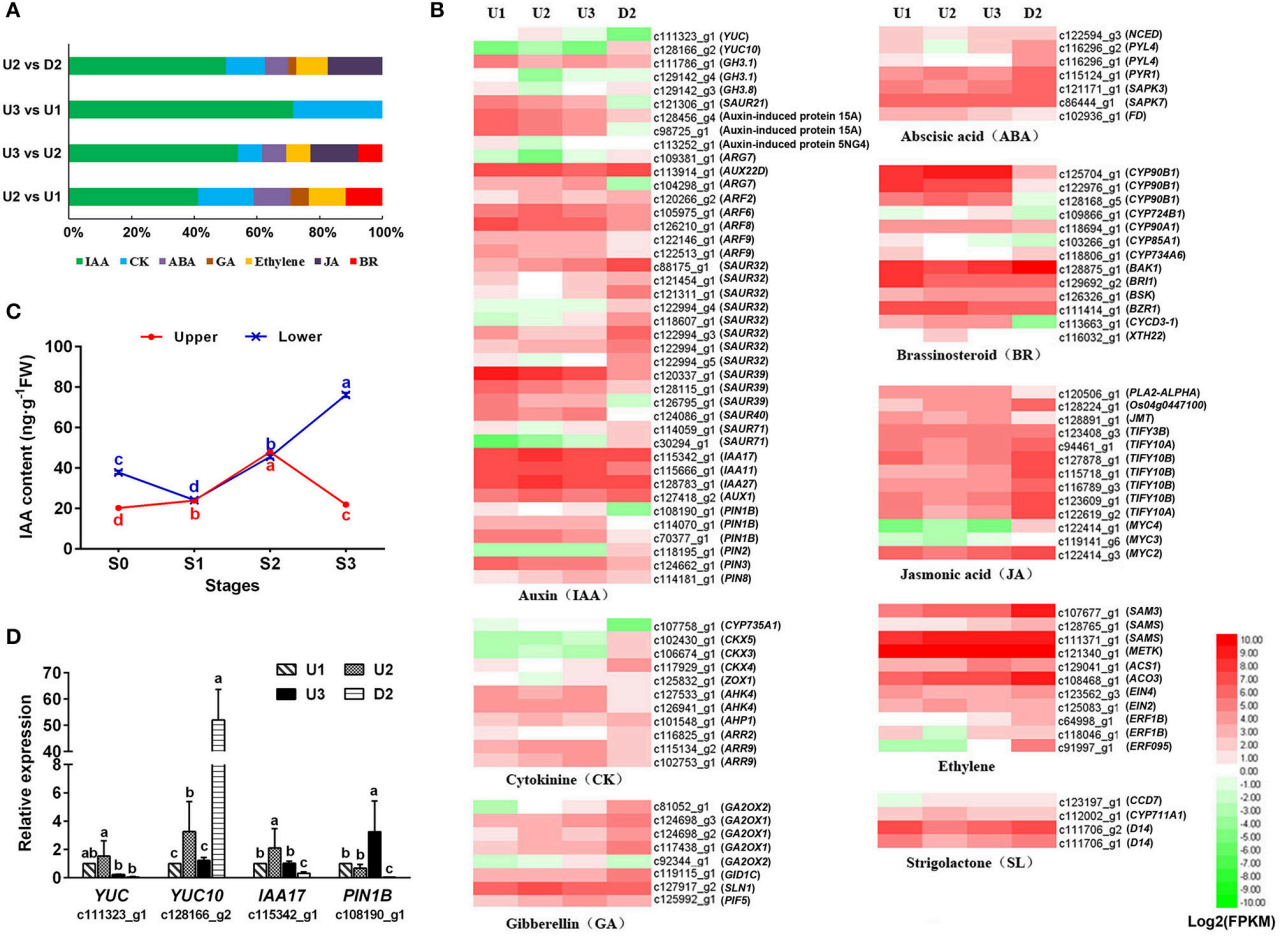
Regulation patterns of plant hormones during the process of bulbil formation. **(A)** Distribution of DEGs related to plant hormone signal transduction compared pairwise among the four libraries. **(B)** Heatmap of the unigenes assigned to hormone synthesis and signal transduction pathways in the four transcriptomes: U1, U2, U3, and D2. The FPKM values of the unigenes were log_2_ transformed, and a heatmap was generated using HemI 1.0. Red and green indicate up- and down-regulated transcripts, respectively. **(C)** IAA contents of the upper (red) and lower (blue) axils at different stages during bulbil formation. The representation of the x-axis, y-axis, significance tests and error bars are as described in Figure 5. **(D)** qRT-PCR analysis of the expression profiles of four genes related to auxin signailing. The representation of the x-axis, y-axis, significance tests and error bars are as described in Figure 4C. Non-overlapping letters (a–d) in **(C,D)** indicate significant differences (*p* < 0.05) for between the comparisons of different samples.

The KEGG enrichment analysis of our transcriptome revealed that a fairly large number of DEGs were assigned to “plant hormone signal transduction” (ko04075) pathways, including auxin (IAA), cytokinin (CK), ethylene, gibberellin (GA), abscisic acid (ABA), brassinosteroid (BR), jasmonic acid (JA) and strigolactone (SL) signaling (Figure 6B, Table S12). IAA and CK signaling were enriched in all pairwise comparisons of the four libraries, with the number of genes related to IAA accounting for the highest percentage (Figure 6A).

During the process of bulbil formation in upper leaf axils of triploid *L. lancifolium*, the IAA content increased from S0 to S2, peaked at S2 (47.95 ng/g FW), and then decreased significantly in S3 (21.95 ng/g FW). In contrast, the IAA content of the lower leaf axils increased continuously from S1 to S3 and was 3.5-fold greater than that in the upper part at S3 (Figure 6C). Forty-one DEGs related to IAA biosynthesis and signal transduction were identified (Table S12). In the IAA biosynthesis pathway, two *YUCCA* genes (c111323_g1 and c128166_g2) were up-regulated in U2 and then down-regulated in U3 during bulbil formation. In the IAA signaling pathway, the *AUX1* (c127418_g2) gene encoding the auxin transporter protein 1 and genes encoding the auxin-responsive protein (*IAA17*_c115342_g1, *IAA11*_c115666_g1, and *IAA27*_c128783_g1) also exhibited increased expression in U2 and reduced expression in U3. *GH3* genes (*GH3.1*_c111786_g1, *GH3.1*_c129142_g4, and *GH3.8*_c129142_g3) encoding indole-3-acetic acid-amido synthetase, which conjugates IAA to amino acids and then reduces IAA concentrations, were down-regulated in U2 vs. U1 and up-regulated in U3 vs. U2. In addition, an auxin efflux carrier component (*PIN1B_* c108190_g1) exhibited the same regulatory pattern as *GH3* genes (Figure 6B). Validation of *YUC* (c111323_g1), *YUC10* (c128166_g2), *IAA17* (c115342_g1), and *PIN1B* (c108190_g1) via qRT-PCR further confirmed our observations (Figure 6D). These results indicated that IAA accumulation in a short time likely promoted bulbil initiation and subsequently inhibited further axillary bulbil formation.

*CYP735A1* (c107758_g1), which catalyzes the biosynthesis of trans-zeatin, was up-regulated during upper bulbil formation and was obviously up-regulated in U2 vs. D2 (Figure 6B, Table S12). Genes (*CKX5*_c102430_g1, *CKX3*_c106674_g1, and *CKX4*_c117929_g1) related to the degradation of CK exhibited increased expression in D2 compared with U1, U2, and U3. In the CK signal transduction pathway, the expression of *AHK4* (c127533_g1 and c126941_g1) and *AHP1* (c101548_g1) was increased in U1, U2, and U3 compared with D2, and the expression level of *AHP* was the highest in U2. These results indicated that the CK signaling pathway likely played a role in promoting bulbil formation in *L. lancifolium*. In addition, BR signaling exhibited a promoting function. *CYCD3-1* (c113663_g1), which functions downstream of BR signaling, was up-regulated during bulbil initiation and formation but showed low expression in D2 (Figure 6B, Table S12).

In the GA biosynthesis pathway, four *GA2OX* (c81052_g1, c124698_g3, c124698_g2, and c117438_g1) genes were slightly up-regulated during bulbil formation, but their expression levels in U1, U2, and U3 were reduced compared with D2. In the GA signaling pathway, *GID1C* (c119115_g1) was down-regulated in U2. *GID1C* levels were increased in D2 compared with U1, U2 and U3. The *SLN1* gene (c127917_g2) encoding the DELLA protein, a repressor of the GA signaling pathway, exhibited a regulatory pattern opposite that of *GID1C* (Table S12). The expression of *D14* (c111706_g2 and c111706_g1) in the SL signaling pathway was the lowest in U2; furthermore, SL potentially inhibits bulbil initiation (Figure 6B, Table S12).

## Discussion

To understand the process of bulbil formation in triploid *L. lancifolium* and to obtain complete transcriptome information during bulbil formation, we first analyzed the anatomical structures of the upper leaf axils, which generate bulbils, and the lower leaf axils, which cannot form bulbils, throughout the period of bulbil formation. Bulbil formation in triploid *L. lancifolium* is a notable illustration of axillary organogenesis, by which axillary cells undergo dedifferentiation to form AM cells on the adaxial side of the petiole base and then differentiate into a bulbil structure (Figures 1E–H) (Grbic and Bleecker, 2000; Wang and Cronk, 2003; Li et al., 2012). During this process, AM of triploid *L. lancifolium* initiated *de novo* from cells in the leaf axil after internodes of stem elongation and leaf unfolding; the same process occurs in *Arabidopsis* (Long and Barton, 2000; Leyser, 2003). In addition, upper axil swelling was observed on the adaxial side of the petiole base at stage S2, which provided a reference for the transcriptome sampling.

Based on the results of histological analysis, three types of samples from leaf axils located on the upper stem (U1, axil; U2, swelling axil; and U3, white dot structure axil) and one from leaf axils located on the lower stem at the stage of swelling in the upper axil (D2) were obtained for RNA-Seq analysis. Unlike other studies on bulbil formation, our analysis was focused not only on bulbil initiation in the leaf axil but also on the reason that bulbils exclusively form on the upper part of the stem in *L. lancifolium*. Approximately 46–70 million raw paired-end reads, with greater than 6.5 Gb per sample, were obtained (Table 1). The results of DEG analysis revealed that bulbil initiation associated with swelling on the adaxial side of the petiole base was particularly important in the process of bulbil formation (Figure 3, Figure S6). Starch and sucrose metabolism and plant hormone signal transduction may play important roles in bulbil formation in triploid *L. lancifolium* (Figures 4, 6).

### Starch synthesis plays a crucial role in bulbil initiation in *L. lancifolium*

Based on the KEGG enrichment analysis, numerous DEGs were found to be enriched in starch and sucrose metabolism pathways, and most of these DEGs were involved in the process of starch synthesis (Table S11). The synthesis of starch in triploid *L. lancifolium* was positively related to the initiation of bulbils, according to the results of RNA-Seq analysis, qRT-PCR verification and starch and soluble sugar determination (Figures 4, 5); this finding is consistent with the swelling of storage organs in potato and *Tulipa edulis* (Geigenberger et al., 1994; Abelenda et al., 2011; Yang et al., 2015; Miao et al., 2016b) and the formation of bulblets from scales in *L. davidii* var. *unicolor* (Li et al., 2014).

In the process of starch synthesis, sucrose serves as the basic metabolic substance and is converted to fructose and UDP-glucose by SUS, generating the preconditions for starch synthesis (Wang et al., 1993). SUS is an exclusive and necessary enzyme for the entry of sucrose into the pathway of sucrose-starch metabolism (Sung et al., 1989). In our study, four of the six *SUS* genes (*SUS2*_c111432_g1, *SUS5*_c126221_g1, *SUS6*_c111800_g1, *SUS7*_c126221_g2) exhibited the highest expression in U2 and the lowest expression in D2 (Figure 4B, Table S11). *SUS* is expressed at high levels in sink tissues, such as potato tubers (Zrenner et al., 1995). During stolon formation in *Tulipa edulis*, the activity of SUS peaks in the initial period, and the contents of soluble sugars and starch are significantly enhanced in the middle period, when the stolons emerge (Miao et al., 2016a). Thus, the up-regulation of SUS may play a key role in bulbil initiation.

In addition, the *AGPS* (*AGPS1*_c121621_g2, *AGPS1*_c121621_g3 and *ADG1*_c120596_g2), *SSS* (*SSS1*_c4952_g1 and *SSS3*_c122034_g2), *GBSS* (*WAXY*_c123636_g2) and *SBE1* (c97206_g1 and c108260_g1) genes exhibited the same up-regulated expression pattern during bulbil initiation (Figures 4B, C). *AGPS* encodes AGPase, which catalyzes the first committed step of starch synthesis in the plastids, converting glucose 1-phosphate (G1P) to ADP-glucose. *SSS, GBSS* and *SBE* are key enzymes in starch synthesis (Tetlow et al., 2004). All of these results indicated that starch synthesis likely promotes bulbil initiation in the early stage of bulbil formation and support the view that starch synthesis can satisfy the demands for reserve substances and energy in active cells, including those related to cell division and differentiation as well as starch accumulation in preparation for the initiation of new plant organs (Miao et al., 2016a).

### Plant hormone signaling exhibits complex regulation during bulbil formation

Our transcriptome analysis identified numerous genes involved in the plant hormone response to bulbil formation (Table S12), and different hormone-associated pathways exhibited distinct regulation patterns (Figure 6B).

Auxin may promoted bulbil initiation and then inhibited the further formation of axil bulbils according to our results. We observed that *YUCCA* genes (c111323_g1 and c128166_g2) were up-regulated in U2 vs. U1 and then down-regulated in U3 (Figures 6B, D). These genes play a key role in the synthesis of IAA; thus, their up-regulated expression is positively related to IAA accumulation (Zhao, 2010). At the same time, *GH3* genes (c111786_g1, c129142_g4, and c129142_g3) and an IAA efflux carrier component (*PIN1B_* c108190_g1), which reduce IAA concentrations, were down-regulated in U2 vs. U1 and then up-regulated in U3. These results are consistent with the notion that IAA accumulation induces new primordia in maize and potato and inhibits the outgrowth of axillary organs in *Arabidopsis* (Greb et al., 2003; Reinhardt et al., 2003; Roumeliotis et al., 2012). However, these results are inconsistent with what occurs in *Agave tequilana* and tomato, which exhibit an inhibitory effect of IAA on the development of new meristems and vegetative bulbils (Wang et al., 2014; Abraham-Juarez et al., 2015). This discrepancy may be caused by the different locations of IAA within plants and differences in IAA concentrations. IAA is largely produced in young expanding leaves at the shoot apex and is transported basipetally down the stem (Ljung et al., 2001; Durbak et al., 2012). Consequently, the concentration of IAA at the shoot apex is higher than in the middle of the stem, and the concentration at the base of stem is the lowest. *L. lancifolium* bulbils first arise at a certain position in the mid-upper leaf axil, instead of at the shoot apex, and the lower stem cannot form bulbils during its life cycle. These findings indicate that bulbil initiation in *L. lancifolium* is related to the concentration of IAA, which can induce bulbil formation after surpassing a certain threshold. However, the exact threshold range is unclear and will be investigated in further studies.

CKs play critical roles in the entire process of bulbil formation. In our study, *CYP735A1* (c107758_g1), which is involved in CK biosynthesis, was obviously up-regulated during bulbil formation in the upper leaf axil. In addition, the expression of *CKX* (*CKX* 5_c102430_g1, *CKX3_* c106674_g1, *CKX4*_c117929_g1) exhibited minimal changes in the upper leaf axil during bulbil formation and was highest in D2 (Figure 6B, Table S12). High CK biosynthesis and low degradation occurred in the upper stem might promote bulbil formation in the upper leaf axil. This finding is closely related to the roles of CKs in regulating cell proliferation and tissue differentiation in many plants (Miller et al., 1955; Hwang et al., 2012). Similar results were reported by Guivarc'h et al. (2002). In addition, a homolog of *CYCD3-1* (c113663_g1), which is induced by CK and plays a key role in integrating cell division and lateral organ development (Riou-Khamlichi et al., 1999), was up-regulated during bulbil formation, and its expression level was lowest in D2. This result further verifies the important role of the promotion of CK in cell division in bulbil formation.

Based on the KEGG analysis, we also identified GA-, SL-, ABA-, ethylene-, and JA-related genes, such as *GID1, D14, SNRK2, PYL4, ERF, JAZ*, and *MYC*. These genes all showed the same expression pattern (Figure 6B): they were down-regulated in U2 vs. U1 and presented the highest expression in D2, indicating an inhibitory effect on bulbil initiation.

Overall, bulbil formation in the upper portion of the stem of triploid *L. lancifolium* is a complex process. Bulbil initiation is particularly important for bulbil formation and might be controlled by genes involved in hormone signal transduction and starch biosynthesis. Further studies on the functions of these candidate genes might be helpful for understanding bulbil formation in lily.

## Author contributions

JM conceived and designed the study. PY performed most of the experiments, with assistance from LX, HX, GH, YF, YC, YT, and SY. The manuscript was written by PY.

### Conflict of interest statement

The authors declare that the research was conducted in the absence of any commercial or financial relationships that could be construed as a potential conflict of interest.
