# Supplementary material for: Histological and Transcriptomic Analysis during Bulbil Formation in Lilium lancifolium
Source: Front Plant Sci. 2017 Aug 30;8:1508. doi: 10.3389/fpls.2017.01508 (PMC5582597; doi:10.3389/fpls.2017.01508)
Supplement: Supplementary file 14 [file Image2.pdf]

Supplementary Material

Histological and Transcriptomic Analysis during Bulbil Formation in *Lilium lancifolium*

Panpan Yang[1,2], Leifeng Xu[2], Hua Xu[2], Yuchao Tang[2], Guoren He[2], Yuwei Cao[2], Yayan Feng[2], Suxia Yuan[2], Jun Ming[1,2*]

[1]College of Landscape Architecture, Nanjing Forestry University, Nanjing, Jiangsu, China

[2]The Institute of Vegetables and Flowers, Chinese Academy of Agricultural Sciences, Beijing, China

*** Correspondence:** Jun Ming: mingjun@caas.cn

Supplementary Figures

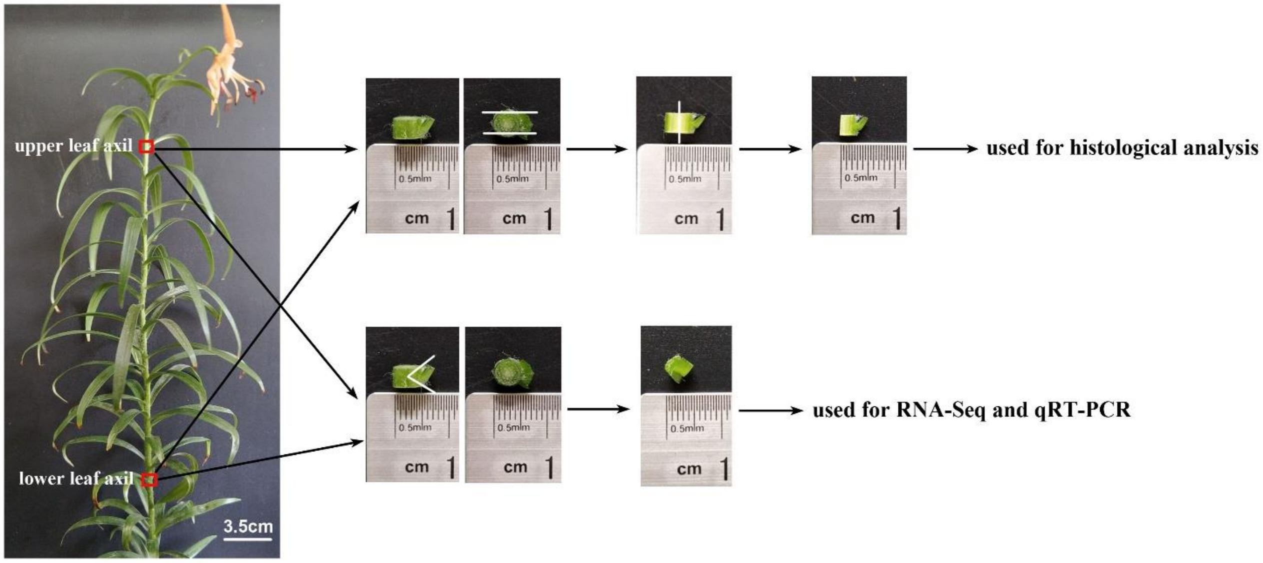

Figure S2. The process of sampling for histology, RNA-Seq and qRT-PCR. Samples were collected by cutting along the white lines.
